# Notes and News

**Published:** 1893-10-14

**Authors:** 


### NOTES AND NEWS.

Collected and Communicated.

The condition of affairs at the Smallpox Hospital, West Ham, revealed through the recent inquiries, is but another instance in confirmation of our views that very great reform is necessary in the management of rate-supported hospitals in this country. The light of public opinion, so freely permitted in the case of our voluntary institutions, is carefully excluded from the Government hospitals, and consequently it is only when a seriously bad condition reaches a climax that any attention is drawn to it. Though reform has commenced, and some institutions are admirably administered, the public do not possess any general guarantee of efficiency in the rate-supported institutions, and the gravest abuses still occur without any effectual check.

Bristol must certainly be regarded as one of our pioneer centres of mental and industrial progress. One local feature of interest is the Industrial and Fine Art Exhibition. This year one section of the exhibition is devoted to the Jenner Collection, which belongs to Mr. Mockler, of Wotton-under-Edge, and has been lent by him. The collection is one of exceptional interest to medical and lay visitors to the exhibition alike. It comprises portraits of Dr. Edward Jenner and of his family by celebrated artists of the day; many curiosities collected by Dr. Jenner himself; his writings in print and manuscript; autograph letters by him and by some of his well-known correspondents; diplomas and certificates received by him; many plates illustrating the course of various cases of small-pox and vaccination; and drawings and documents by members of his family, or relating to them, and the written works of the celebrated physician exist here in a very complete form. A most comprehensive and interesting catalogue, to be obtained at the exhibition, enhances the interest of the collection. It is prefaced by a short memoir of the great discoverer, and proceeds to give short but full information of each object on view. Some curious correspondence is to be seen between Dr. Jenner and a French doctor on the subject of a release of or exchange between French and English prisoners of war, besides other interesting letter. In short, the collection is a distinct "find" on the part of the Bristol Exhibition, and we hope that other centres will some day also be able to offer the same opportunity of a sight which visitors to Bristol have recently enjoyed.

The Duke and Duchess of York in visiting Edinburgh recently did not forget the hospitals. The Royal Infirmary and the Longmore Hospital were both visited, and met with the warm approbation of the Royal visitors. At the Longmore the new wing was opened by their Royal Highnesses, who stated that the Queen had given her permission that the institution should be entitled the Royal Association for Incurables.

A Hospitals' Exhibition is in course of development for next year. We understand that its site will be in Woodhouse Park, Uxbridge Road, and confident hopes are entertained by its originators that it will be popular and successful. Experience of similar ventures, however, does not inspire us with much confidence and efforts of this kind often prove a loss rather than a blessing to the institutions. A loss of credit is almost a worse evil than a shortness of cash. The proceeds are to be devoted to the needs of the various Metropolitan hospitals. Major Wyndham Malet, honorary director of the late Military Exhibition, is one of the chief movers in the scheme.

We cannot agree with the Hospital Saturday Fund in endeavouring to throw responsibility on the hospitals themselves for the falling off in the street collections. They argue that collections of a like kind adopted by the institutions, as well as by the Hospital Saturday Fund, weaken the cause by giving a distaste for such excessive importunity. It is but natural that any method upheld and found successful by so important a body as the Fund should be used by individual charities too. But the public are getting wiser, and experience shows that boxes are an unworthy and costly means of collecting. They offer the maximum of opportunity for fraud when scattered over a vast area like London. The frauds are of two kinds—direct, as at Norwood—and we have no evidence that such frauds are not spread over the whole system—and indirect, arising from spurious collectors who haunt the public streets. In these circumstances the action of the Council of the Hospital Saturday Fund in virtually censuring by implication those who have brought the recent frauds to light, is calculated to excite public indignation against themselves. We propose to show next week how the London system has made these frauds possible.

As the result of experience, we would advise the abolition of boxes in connection with the Hospital Saturday Fund Collection. This plan has been tried at Leeds, where the Fund has had the courage to refuse to be identified with street collections, with complete success, the harvest reaped showing a material increase, whereas that of London is not progressing satisfactorily at all. We are inclined to think it would be wise for hospital committees to decline all further grants from the Hospital Saturday Fund unless the Council see their way to abolish the street collection altogether. Street collections have made the metropolitan hospitals unpopular; have irritated and estranged liberal supporters of hospitals, and dwarfed in the public eye the importance of large contributions to hospitals and the excellent business methods which, on the whole, characterise their management. It passes our comprehension how Mr. Acland and his Council can continue to sanction this bad and improper system, which tends towards the steady diminution of hospital revenues despite the few miserable pounds which the boxes gather.

